# ITGB2-mediated metabolic switch in CAFs promotes OSCC proliferation by oxidation of NADH in mitochondrial oxidative phosphorylation system

**DOI:** 10.7150/thno.47901

**Published:** 2020-10-26

**Authors:** Xiaoxin Zhang, Yingchun Dong, Mengxiang Zhao, Liang Ding, Xihu Yang, Yue Jing, Yuxian Song, Sheng Chen, Qingang Hu, Yanhong Ni

**Affiliations:** 1 Central Laboratory, Nanjing Stomatological Hospital, Medical School of Nanjing University, 30 Zhongyang Road, Nanjing 210008, China.; 2 Department of Anesthesiology, Nanjing Stomatological Hospital, Medical School of Nanjing University, Nanjing 210008, China.; 3 Department of Oral & Maxillofacial Surgery Nanjing Stomatological Hospital, Medical School of Nanjing University, 30 Zhongyang Road, Nanjing 210008, China.; 4 Department of Oral Pathology, Nanjing Stomatological hospital, Medical School of Nanjing University, Nanjing, Jiangsu 210008, China.

**Keywords:** oral squamous cell carcinoma, cancer associated fibroblasts, ITGB2, lactate, NADH, oxidative phosphorylation system

## Abstract

**Objectives:** Integrins, the coordinator of extracellular and intracellular signaling, are often found to be aberrant in tumors and can reshape the tumor microenvironment. Although previous studies showed that integrin beta 2 (ITGB2) is important for host defense, its expression profile and role in tumors, especially in cancer associated fibroblasts (CAFs) are still unknown.

**Methods:** Immunofluorescence stain and fluorescence activated cell sorting were used to analyze the ITGB2 expression profile in oral squamous cell carcinoma (OSCC). RT-PCR and western blot were used to compare ITGB2 expression in normal fibroblasts (NFs) and cancer associated fibroblasts (CAFs). Clinical data and function-based experiments were used to investigate the promoting tumor growth ability of ITGB2 expressing CAFs. Enhanced glycolysis activity was identified by using bioinformatics analyses and GC/MS assays. MCT1 knockdown OSCC cell lines were constructed to explore the pro-proliferative mechanisms of ITGB2 expressing CAFs in multiple *in vitro* and *in vivo* assays.

**Results:** We found that CAFs exhibited significantly higher ITGB2 expression than the matched NFs. In addition, higher ITGB2 expression in CAFs was correlated with higher TNM stages and more Ki67+ tumor cells, indicating its ability to promote OSCC proliferation. Further, co-culture assay demonstrated that ITGB2-mediated lactate release in CAFs promoted OSCC cell proliferation. Mechanically, ITGB2 regulated PI3K/AKT/mTOR pathways to enhance glycolysis activity in CAFs. Accordingly, lactate derived from ITGB2-expressing CAFs was absorbed and metabolized in OSCC to generate NADH, which was then oxidized in the mitochondrial oxidative phosphorylation system (OXPHOS) to produce ATP. Notably, inhibiting the OXPHOS system with metformin delayed the proliferative capacity of OSCC cells cultured in the ITGB2-expressing CAFs medium.

**Conclusions:** Our study uncovered the ITGB2^high^ pro-tumoral CAFs that activated the PI3K/AKT/mTOR axis to promote tumor proliferation in OSCC by NADH oxidation in the mitochondrial oxidative phosphorylation system.

## Introduction

Oral squamous cell carcinoma (OSCC) is the most common form of carcinoma in the oral cavity and ranks as the 12^th^ most common cancer worldwide 1. Despite its improved treatment, the overall survival rates still hover around 47% and pose a serious threat to overall health worldwide 2. Recently, the crosstalk between cancer cells and the surrounding microenvironment has been found to contribute to tumor growth and metastasis and now is a promising therapeutic target. Cancer associated fibroblasts (CAFs), the second most abundant cell type in the tumor microenvironment, are found to be implicated in poor clinical outcomes for patients with OSCC and several other tumor types 3-6.

CAFs promote tumor growth, survival and metastasis by remodeling the extracellular matrix, reshaping the tumor immune microenvironment and reprogramming tumor metabolism 4, 7. Among them, the metabolic reprogramming of CAFs reflects their adaption to tumor energy requirements, as metabolic change is one of the six hallmarks of cancer. However, CAFs display different metabolic characteristics because of metabolic heterogeneity between tumors 8. For example, in breast cancer, prostate cancer and melanoma, the CAFs-mediated reverse Warburg effect, where CAFs undergo anaerobic glycolytic activity to release lactate to nearby tumor cells, has been reported 9-13. However, in ovarian cancer CAFs mediated anaerobic glycolysis in tumor and released lactate to CAFs *via* LINC00092 14. These contradictory findings indicated that the reciprocal metabolic change between CAFs and cancer cell is far more complicated than we previously anticipated. Therefore, it is important to explore the mechanism of the CAFs-mediated metabolic switch to identify subtype-selective metabolic vulnerabilities.

It is now emerging that NADH levels are associated with cancer progression and subsequently oxidative phosphorylation systems (OXPHOS) are essential for cancers 15. Santidrian and Wang *et al.* found that decreased NAD^+^/NADH levels rendered tumor cells more aggressive and increased metastasis 16. In addition, Zhao *et al.* showed that increasing the NAD^+^/NADH ratio by using chemical compounds led to cell death 17. However, how NAD^+^/NADH is regulated by microenvironmental cues is unknown.

Integrins are a family of cell-surface glycoproteins that sense the extracellular matrix and trigger a range of cellular responses to control cell adhesion, migration, proliferation, survival and differentiation 18. Recent evidence has shown that integrins also control cell metabolism 19. As they are important for a range of physiological functions, these receptors also play an essential role in promoting a more malignant tumor cell phenotype in cancer. Integrin beta 2 (ITGB2), one of integrin subunits, was previously found to be exclusively expressed in leukocytes. It promotes leukocyte adhesion to the endothelium and the ensuing extravasation 20, 21. However, its expression and functions in CAFs have not previously been discovered. In the present study we found that ITGB2 was upregulated in CAFs than in the matched NFs in OSCC and was associated with poor clinical characteristics and outcomes. Mechanically, ITGB2 promoted glycolysis *via* PI3K/AKT/mTOR pathways in CAFs and secreted lactate to promote OSCC proliferation by enhancing mitochondrial OXPHOS capacities. Using metformin to target the mitochondrial complex I system could effectively inhibit the pro-proliferative effects of ITGB2-expressing CAFs.

## Material and Methods

### Cell culture

CAFs and NFs were obtained from tumor samples and the matched adjacent non-tumor tissues of patients with OSCC treated at the Nanjing Stomatological Hospital, Medical School of Nanjing University as previously described 5, 6. CAFs, which were demonstrated in our previous study, were characterized by the expression of α-SMA and the lack of expression of CK and maintained in DMEM/F-12 supplemented with 10% FBS in a humidified 37 °C incubator with 5% CO_2_ and cultured fewer than 12 passages to ensure biologic similarity to the original specimens. HSC3, SAS, CAL27, SCC9, SCC131 and CAL33 cells were cultured in DMEM supplemented with 10% FBS in a humidified 37 °C incubator with 5% CO_2_.

### Reagents

ITGB2 (#73663), GluT4 (#2213), Akt (#4691), Phospho-Akt (#4060), PI3Kinase (#4292), Anti-rabbit IgG, HRP-linked Antibody (#7074), Anti-mouse IgG and HRP-linked Antibody (#7076) were obtained from Cell Signaling Technology. ITGB2 (ab53009), Phospho-PI3Kinase (ab182651) and a-SMA (ab5694) were obtained from the Abcam company. ITGB2 (10554-1-AP), HIF1a (66730-1-Ig), MCT1 (20139-1-AP), MCT4 (22787-1-AP), LDHB (14824-1-AP) and PDK4 (12949-1-AP) were obtained from the Proteintech company. ITGB2 (AF1730) was purchased from R&D. Secondary anti-rabbit IgG Dylight 680 (#35568), anti-rabbit IgG Dylight 488 (#35553) and anti-mouse IgG Dylight 800 (#35521) were obtained from ThermoFisher.

The primer sequences used in this study were obtained from commercial sources and are displayed in Table S1.

### Immunoblotting

The cell lysates were acquired by scraping the cultured cells and were lysed in RIPA lysis buffer with a mixture of protease and phosphatase inhibitors on ice. Equal amounts of proteins were separated through SDS-PAGE and blocked in 3% BSA. After incubation with primary antibody at 4 °C overnight and HRP conjugated secondary antibody for 1 h, protein bands were detected by the protein imaging system (Tanon5200). Immunoblots for the presented data were analyzed from at least 3 experimental repeats.

### Immunofluorescence (IFC) stain

For cell immunofluorescence, cells mounted on cover glasses were fixed by 4% paraformaldehyde and blocked in 5% BSA for 1 h. The cells were then incubated with primary antibody at 4 °C overnight and dye light conjugated antibodies for 1 hour. The images were acquired with the Nikon Ti Eclipse Confocal Microscope. For frozen immunofluorescence, the slides were fixed in 4% paraformaldehyde and then treated with the procedures described above.

### RNA analysis

RNA was obtained using Trizol reagent following the manufacturer's procedure and then reversed into cDNA using HiScript III RT SuperMix (Vazyme Biotech Co., Ltd). The relevant expression of the genes was determined *via* AceQ® qPCR SYBR® Green Master Mix (Vazyme Biotech Co., Ltd).

### Tissue samples

Samples and the follow-up data of 80 OSCC patients who received cancer surgery from 2008 to 2010 were obtained from the head and neck tumor biobank of Nanjing Stomatological hospital, Medical School of Nanjing University. All patients had received follow-up examinations every two months from the date of surgery to the date of disease progression after surgery until death. The study has been approved by the ethics committee for clinical study of Nanjing Stomatological Hospital.

### Flow cytometry

To isolate CAFs and leucocytes, the tumors from patients with OSCC were digested and dissociated using the gentleMACS™ Dissociator to obtain a single-cell suspension and then incubated with PE conjugated anti-ITGB2 antibody and FITC conjugated anti-CD45 antibody for 30 min. After washing three times with PBS, the cells were sorted by Sony Sorting Flow Cytometer.

To detect membrane ITGB2, CAFs were trypsinized and incubated in Staining Buffer containing APC conjugated anti-CD18 antibody for 30 min and then analyzed using the BD FACSCalibur Flow Cytometer. To detect the total ITGB2 in flowcytometry, the cells were fixed and permeabilized by using the Intracellular Fixation & Permeabilization Buffer Set and then treated with the procedures described as above.

### Immunohistochemistry (IHC) analysis

Sections of formalin-fixed and paraffin-embedded tissues were deparaffinized and subjected to antigen retrieval using 10 mM citrate buffer (92 °C for 30 min). Gene expression was evaluated according to stain intensity and the percentage of positive cells. The intensity of staining was graded as 1 = weak staining, 2 = moderate staining and 3 = strong staining. The percentage of stained cells was graded as 0 = 0-5%, 1 = 6-25%, 2 = 26-50%, 3 = 51-75% and 4 = 75-100%. The final score was obtained by multiplying the two scores. All scorings were conducted by two pathologists without knowledge of the patients' clinical characteristics or outcome.

### Plasmid construction and lentivirus infection

siRNA targeting ITGB2, MCT1, MCT4 and LDHB were cloned into pLVX-Puro, i.e. pLVX-shITGB2, pLVX-shMCT1, pLVX-shMCT4 and pLVX-shLDHB. The full length of the cDNA sequences of ITGB2 was cloned into lentivirus vector pCDH-CMV, i.e. pCDH-ITGB2.

Plasmids were transfected into 293T cells *via* Polyjet and the supernatants were collected 2 days after transfection. After being filtered through a 0.45-μm PES filter, the viral supernatants were either directly added to CAFs or NFs or stored at -80 °C.

### Gas chromatography-mass spectrometry untargeted analysis

When cells were 80% to 90% confluent, cells were collected in 10-cm dishes and sent to Dr. Tong Xie (Nanjing University of Chinese Medicine). Materials and reagents for GC-MS analysis were prepared according to the previous method 22. GC-MS analysis was performed by using a Trace 1310 Gas Chromatograph equipped with an AS1310 auto sampler which connected to a TSQ 8000 triple quadrupole mass spectrometer (Thermo Scientific, Waltham, MA).

### Lactate and glucose assessment

The cell culture supernatants were collected two days after seeding. The supernatants were assessed by using an L-lactate assay kit (EFLLC-100) and glucose assay kit (DIGL-100) according to the manufacturer's instructions.

### Xenografts

Four-week-old male NOD/SCID mice were housed and maintained in laminar flow cabinets under specific pathogen-free conditions. 1) HSC3-Ctrl (1×10^6^ cells) & CAFs-Ctrl (5×10^5^ cells), HSC3-Ctrl (1×10^6^ cells) & CAFs-ITGB2 (5×10^5^ cells), HSC3-shMCT1 (1×10^6^ cells) & CAFs-Ctrl (5×10^5^ cells), HSC3-shMCT1 (1×10^6^ cells) & CAFs-ITGB2 (5×10^5^ cells) were injected into the flanks of nu/nu mice, respectively. Tumor volume was measured with calipers in two dimensions, and volumes were estimated using the equation V = Length × Width × Width/2.

### Public database and GSEA

Gene expression analyses of human tumor stroma were retrieved from NCBI Gene Expression Omnibus (GEO), including GSE40595 and GSE26910. Array data were analyzed using the Gene-set enrichment analysis software [Broad Institute of MIT and Harvard (USA)] according to the program guidelines.

### Proliferation rates

Cells were plated in triplicate in 96-well plates, with an initial seeding density of 3,000 cells. The medium was then changed to CAFs-conditioned medium (CM) which was boiled at 95 °C for 15 min or filtered through a 3-kDa cutoff filter after cell adhesion. After 24 h incubation, the medium was changed to DMEM supplemented with 10% FBS and 10 μL CCK8 solution. The plates were then measured using a microplate reader at 450 nm. The proliferation rate was calculated based on the following formula: [(*As*-*Ab*)/(*Ac*-*Ab*)] ×100%, where *As* is the absorbance of tested wells, *Ac* is the absorbance of control wells and *Ab* is the absorbance of blank wells.

### ATP, NADH and mitochondrial complex I activity quantitation

ATP, NADH and mitochondrial complex I were measured using the specific kit (ATP kit: Beyotime S0026, NADH kit: Beyotime S0175, mitochondrial complex I activity: Abcam ab109903) following the manufacturer's instructions.

### Data analyses

Results are expressed as the means ± standard deviation (s.d.) from at least three independent experiments. Single comparisons between two groups were determined by the Student* t* test. Comparisons between multiple groups were determined by one-way ANOVA followed by the Tukey post-test. *p* values <0.05 were considered significant.

## Results

### ITGB2 is upregulated in CAFs in OSCC tumor microenvironment

**Figure 1:**
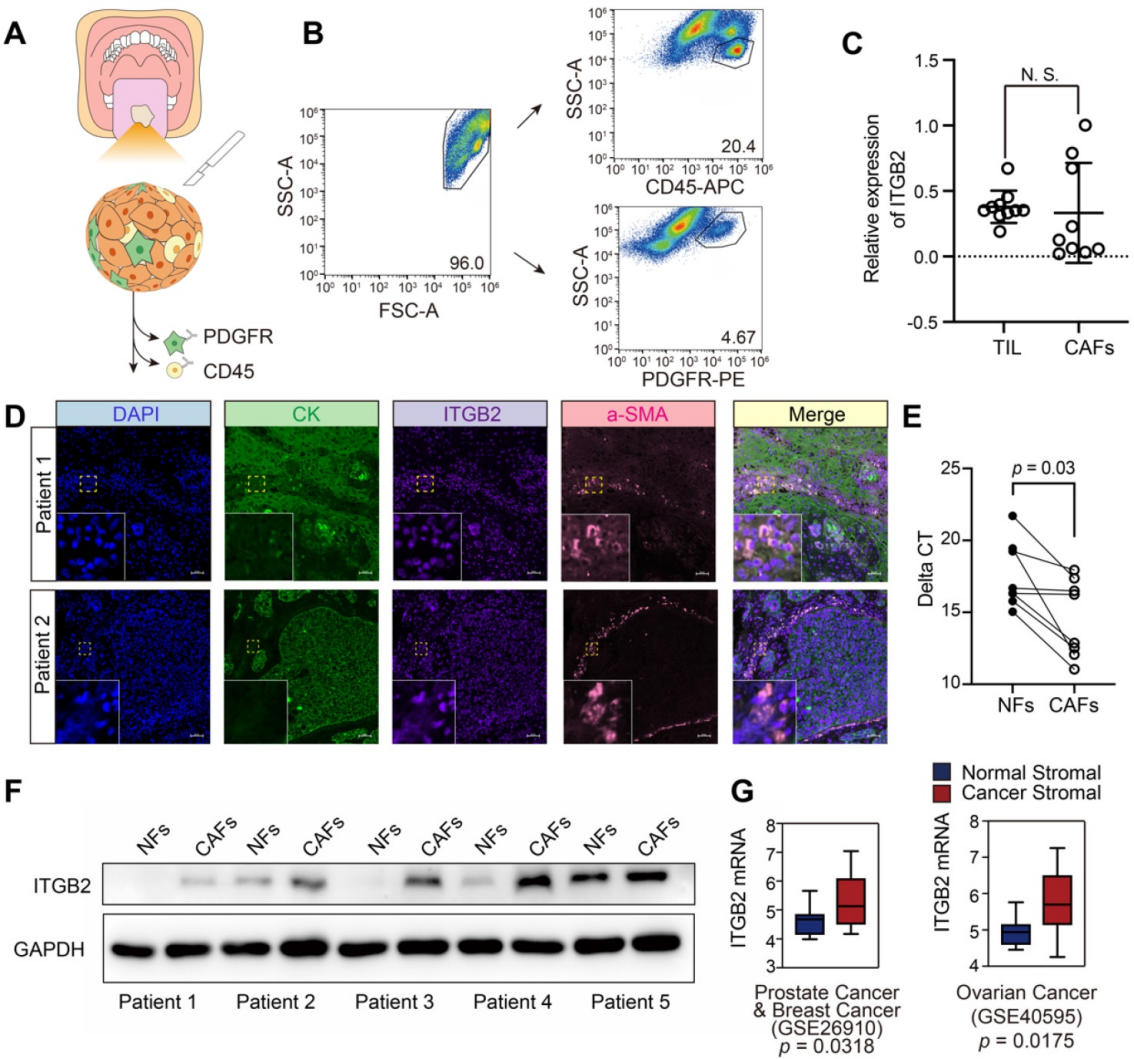
** ITGB2 is upregulated in CAFs derived from OSCC tumor sample than in NFs. A)** Cartoon describing the fluorescence activated cell sorting strategy. **B)** Representative flow cytometry dot plots showing sorting strategy for TIL (CD45^+^) and CAFs (PDGFR^+^) cells. **C)** ITGB2 mRNA expression levels (relative to Gapdh) in TIL (CD45^+^) and CAFs (PDGFR^+^), N=5. **D)** Representative immunofluorescence images of CK (green), ITGB2 (purple), a-SMA (red) in two OSCC patients. Scale bar: 50 µm. **E)** Delta CT values of ITGB2 (relative to Gapdh) in NFs and CAFs. N=5. **F)** Western blot showing protein levels of ITGB2 and GAPDH of 5 paired NFs and CAFs from OSCC patients. **G)** Tukey boxplots showing z-score values of ITGB2 mRNA expression in normal and cancerous stroma from prostate, breast and ovarian cancers. Prostate: normal=6, cancer=6. Breast: normal=6, cancer=6. Ovarian: normal=8, cancer=31. Where indicated, individual *p* values are shown; alternatively the following symbols were used to describe statistical significance: n.s., non-significant.

Since CAFs promote OSCC progression, RNA-sequencing was performed to identify different gene expression profiles between CAFs and the matched NFs from 5 patients with OSCC. Among the most significant upregulated genes, ITGB2, which was reported to be exclusively expressed in leukocytes, was found to be overexpressed in CAFs and caught our attention (Table S2). To confirm its expression profiles in the OSCC tumor microenvironment, we isolated tumor infiltrating immune cells (TILs) and CAFs using CD45 and PDGFRb antibodies by flow cytometry (Fig. 1A-B). RT-PCR results showed no significantly different ITGB2 mRNA levels between CAFs and immune cells, indicating that ITGB2 was not restricted to immune cells and was also expressed in CAFs (Fig. 1C). In addition, IFC analysis of ITGB2 in the OSCC tumor microenvironment also showed that ITGB2 expression was positively correlated with CAFs markers, α-SMA, further confirming ITGB2 expression in CAFs (Fig. 1D).

To confirm the RNA sequence results of CAFs and NFs, 5 pairs of CAFs and NFs were collected. Both RT-PCR and western blot analyses showed that ITGB2 expression levels were significantly upregulated in CAFs compared with those of the matched NFs, indicating its high expression in the OSCC stroma (Fig. 1E-F and Fig. S1). In addition, the investigation of stromal gene expression in normal and cancerous tissues showed that ITGB2 was also significantly upregulated in some other types of tumor stroma, including prostate cancer, breast cancer and ovarian cancer, indicating ITGB2 upregulation in CAFs was not restricted to OSCC stroma (Fig. 1G). Meanwhile, the location of ITGB2 in CAFs was measured by flowcytometry and IFC. Both showed that ITGB2 was not exclusively expressed in membranes of CAFs but was also detected in cytoplasm. Our results indicated that the expression pattern of ITGB2 in CAFs differed from leukocytes but was similar to cancer cells where ITGB2 was also discovered in cytoplasm 23 (Figs. S2A, S2B).

### Higher ITGB2 expression in CAFs correlates with poor clinical characteristics and outcomes of OSCC patients

**Figure 2:**
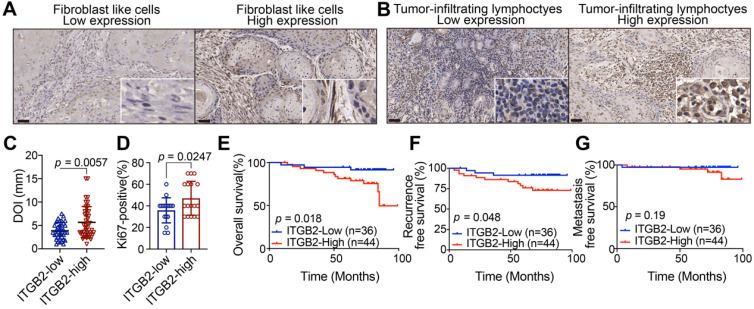
** Higher ITGB2 expression in CAFs correlates with poor clinical characteristics and outcomes in OSCC patients. A)** Representative IHC images of ITGB2 in fibroblast-like cells (FLCs). **B)** Representative IHC images of ITGB2 in tumor-infiltrating lymphocytes (TILs). **C)** Quantification of DOI in ITGB2-low and ITGB2-high CAFs group. N (ITGB2-low group) =35, N (ITGB2-high group) =44. **D)** Quantification of Ki67-positive cells in ITGB2-low and ITGB2-high CAFs group. N (ITGB2-low group)=17, N (ITGB2-high group)=17. E-G) Kaplan-Meier survival curves of overall survival **(E)**, recurrence-free survival **(F)** and metastasis free survival **(G)** for OSCC patients according to ITGB2 expression levels in CAFs. Individual *p* value was shown in each figure.

IHC analysis of ITGB2 was further performed in 80 patients with OSCC. Results showed that ITGB2 was widely distributed throughout the tumor microenvironment, including cancer cells, TILs and CAFs (Fig. 2A-B). As ITGB2 was expressed both in immune cells and CAFs, we then investigated the diagnostic value of ITGB2 expression in CAFs and TILs. Table 1 showed that higher ITGB2 expression in TILs was only associated with older age. On the contrary, its higher expression in CAFs was associated with higher TNM and deeper DOI (Depth of invasion) (Table 1, Fig. 2C). In addition, higher ITGB2 expression in CAFs was accompanied by increased Ki67 positive cells (Fig. 2D), while no correlation between ITGB2 expression in TILs and DOI was found (Table 1). Kaplan-Meier analysis further revealed that patients with higher ITGB2 expression had shorter survival and earlier recurrence (Fig. 2E, F). The above results revealed that that aberrant ITGB2 expression in CAFs was correlated with poor clinical characteristics and outcomes, indicating that ITGB2^hi^ CAFs may promote OSCC cell proliferation.

### ITGB2-expressing CAFs might promote OSCC cell proliferation *via* metabolites

**Figure 3:**
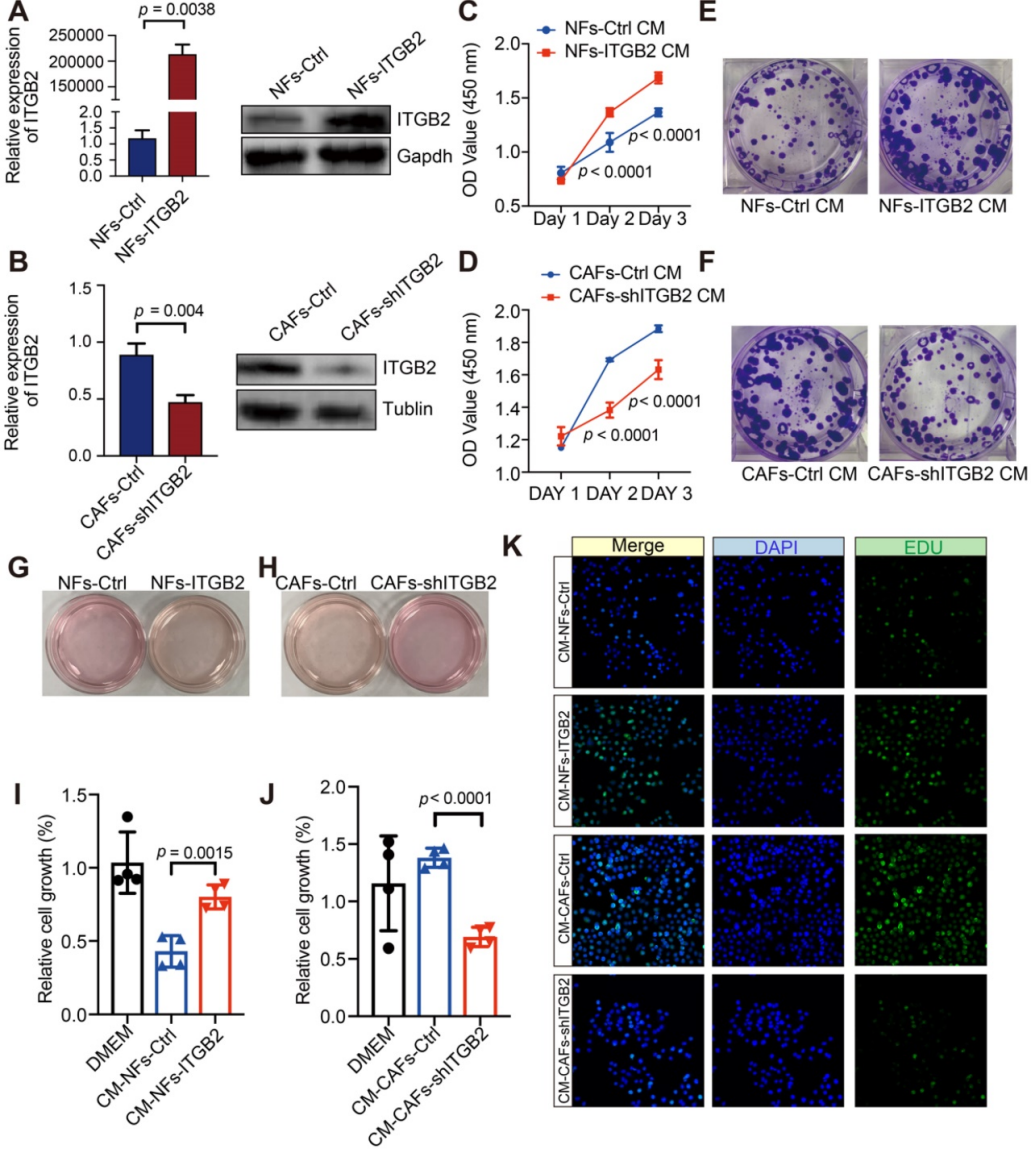
** ITGB2-expressing CAFs promote OSCC proliferation not *via* cytokines. A)** Relative expression of ITGB2 between NFs-Ctrl and NFs-ITGB2 by RT-PCR (Left) and western blot (Right); **B)** Relative expression of ITGB2 between CAFs-Ctrl and CAFs-shITGB2 by RT-PCR (Left) and western blot (Right). **C)** Proliferation of HSC3 cell at indicated time after culturing in NFs-Ctrl or NFs-ITGB2 cultured medium. N=3. **D)** Proliferation of HSC3 cell at indicated time after culturing in CAFs-Ctrl or CAFs-shITGB2 cultured medium. N=3. **E)** Representative images of colonies formed in NFs-Ctrl and NFs-ITGB2 cultured medium. **F)** Representative images of colonies formed in CAFs-Ctrl and CAFs-shITGB2 cultured medium. **G-H)** Color of medium of NFs-Ctrl versus NFs-ITGB2 (G) and CAFs-Ctrl versus CAFs-shITGB2 (H). **I)** Proliferation of HSC3 cells at 24 hours post culturing in CM-NFs-Ctrl or CM-NFs-ITGB2. N=4. **J)** Proliferation of HSC3 cells at 24 hours post culturing in CM- CAFs-Ctrl or CM-CAFs-shITGB2. N=4. **K)** Representative immunofluorescence images of EdU (Green) incorporation into HSC3 at 24 hours post culturing in CM-NFs-Ctrl, CM-NFs-ITGB2, CM-CAFs-Ctrl or CM-CAFs-shITGB2. Individual *p* value was shown in each figure.

To explore the function of ITGB2 in CAFs, NFs for ITGB2 overexpression experiments and CAFs for ITGB2 knockdown experiments were performed, and HSC3 cells were then cultured in a fibroblasts-cultured medium (Fig. 3A-B). When ITGB2 was overexpressed in NFs, the growth rates of OSCC were significantly upregulated, while its growth rates were significantly decreased when ITGB2 was silenced in CAFs (Fig. 3C-D). Similarly, the colony forming number of HSC3 increased when ITGB2 was overexpressed and decreased when ITGB2 was silenced (Fig. 3E-F), indicating ITGB2-expressing CAFs promoted tumor cell proliferation.

Interestingly, we noticed that the color of the NFs-ITGB2 culture medium was yellow, while that of the NFs-Ctrl was pink (Fig. 3G). Similar results were also found in CAFs when ITGB2 was silenced (Fig. 3H). To further prove that the acidic catabolites promoted OSCC proliferation, we seeded HSC3 in heated fibroblasts cultured medium, i.e. CM, in which all proteins had been denatured and lost function. As shown in Fig. 3I and 3J, even if the medium was heated, CM-NFs-ITGB2 still promoted OSCC proliferation, and knocking down ITGB2 in CAFs affected HSC3 proliferation. In addition, the pro-proliferative effects of CM-CAFs were abrogated by adding ITGB2 neutralizing antibody (Fig. S3). We then performed an EDU cell proliferation assay of HSC3 in CM. Similarly, EDU-positive cells in CM-NFs-ITGB2 increased markedly more than in CM-NFs-Ctrl and decreased more in CM-CAFs-shITGB2 than in CM-CAFs-Ctrl (Fig. 3K). Moreover, after the medium had passed through a 3 kDa cutoff filter that removed molecules larger than 3 kDa, the ITGB2 expression level-dependent ability to regulate cell growth was retained (Fig. S4A, S4B). Taken together, the culture medium of ITGB2 expressing CAFs contained small molecules (< 5 kDa) that withstood high temperature, indicating the molecules could be metabolites.

### Glycolytic activity is enhanced in ITGB2-expressing CAFs

**Figure 4:**
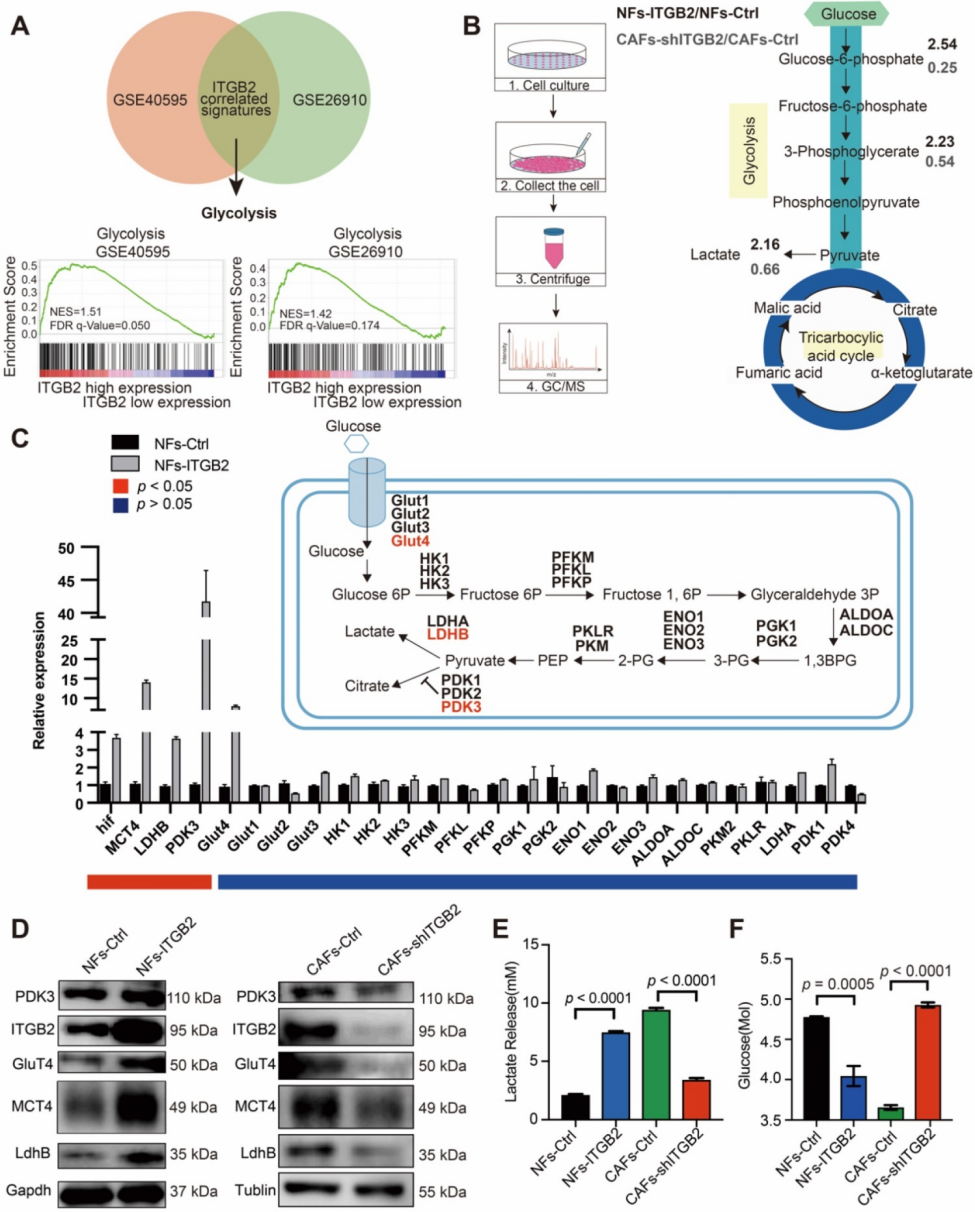
** ITGB2 regulates glycolysis in CAFs. A)** GSEA plot of the association between gene sets positively correlated with ITGB2 in the stroma profile of GSE40595 and GSE26910. **B)** Left: Cartoon describing the experimental setup for LC-MS analyses of NFs-Ctrl, NFs-ITGB2, CAFs-Ctrl, CAFs-shITGB2. Right: Graphical presentation of LC-MS results by flow diagram, showing glycolytic metabolite ratios between NFs-Ctrl versus NFs-ITGB2 (marked in bold black numbers) or CAFs-Ctrl versus CAFs-shITGB2 (marked in bold grey numbers). **C)** mRNA gene expression levels (relative to Gapdh) of the glycolytic enzymes in NFs-Ctrl and NFs-ITGB2. N=2. **D)** Western blot showing the proteins regulating glycolysis, gapdh and tublin between NFs-Ctrl versus NFs-ITGB2 (Left) and CAFs-Ctrl versus CAFs-shITGB2 (Right). **E)** Lactate levels in medium of NFs-Ctrl, NFs-ITGB2, CAFs-Ctrl and CAFs-shITGB2 at 24 hours. N=3. **F)** Glucose levels in medium of NFs-Ctrl, NFs-ITGB2, CAFs-Ctrl and CAFs-shITGB2. N=3. Individual *p* value was shown in each figure.

In order to explore the biological mechanism that explains the elevated acidic catabolites accompanied by ITGB2 upregulation in CAFs, GSEA was performed using GSE40595 and GSE26910 data. Both databases showed that the gene set of HALLMARK_GLYCOLYSIS was highly enriched in the ITGB2-high group (Fig. 4A), which indicated that ITGB2 was involved in glycolysis in CAFs. Consistently, GC-MS analyses of NFs-Ctrl, NFs-ITGB2, CAFs-Ctrl and CAFs-shITGB2 cells showed that some glycolytic metabolite ratios increased between NFs-ITGB2 and NFs-Ctrl, and decreased between CAFs-shITGB2 and CAFs-Ctrl, such as glucose-6-phosphate, 3-phosphoglycerate and lactate (Fig. 4B).

To determine whether ITGB2 mediated glycolytic activity in fibroblasts, we measured the glycolytic gene expression in NFs-Ctrl, NFs-ITGB2, CAFs-Ctrl and CAFs-shITGB2. Both western blots and RT-PCR showed that the genes regulating anaerobic glycolysis, i.e. HIF1a, MCT4, LDHB, PDK3 and Glut4, were overexpressed in the NFs-ITGB2 group and down regulated in the CAFs-shITGB2 group (Fig. 4C and 4D). Accordingly, NFs-ITGB2 showed an increase in extracellular lactate levels and a drop in glucose levels compared with NFs-Ctrl. Correspondingly, less lactate and more glucose were also found in the CAFs-shITGB2 group compared with the CAFs-Ctrl group (Fig. 4E and 4F). In addition, glycolytic proteins and lactate were also downregulated in CAFs treated with ITGB2 neutralizing antibody (Fig. S5A, S5B). All these results suggested ITGB2 regulated glycolysis activity in CAFs, but the mechanisms still need to be explored.

### ITGB2 regulates glycolysis *via* PI3K/AKT/mTOR pathways in CAFs

**Figure 5:**
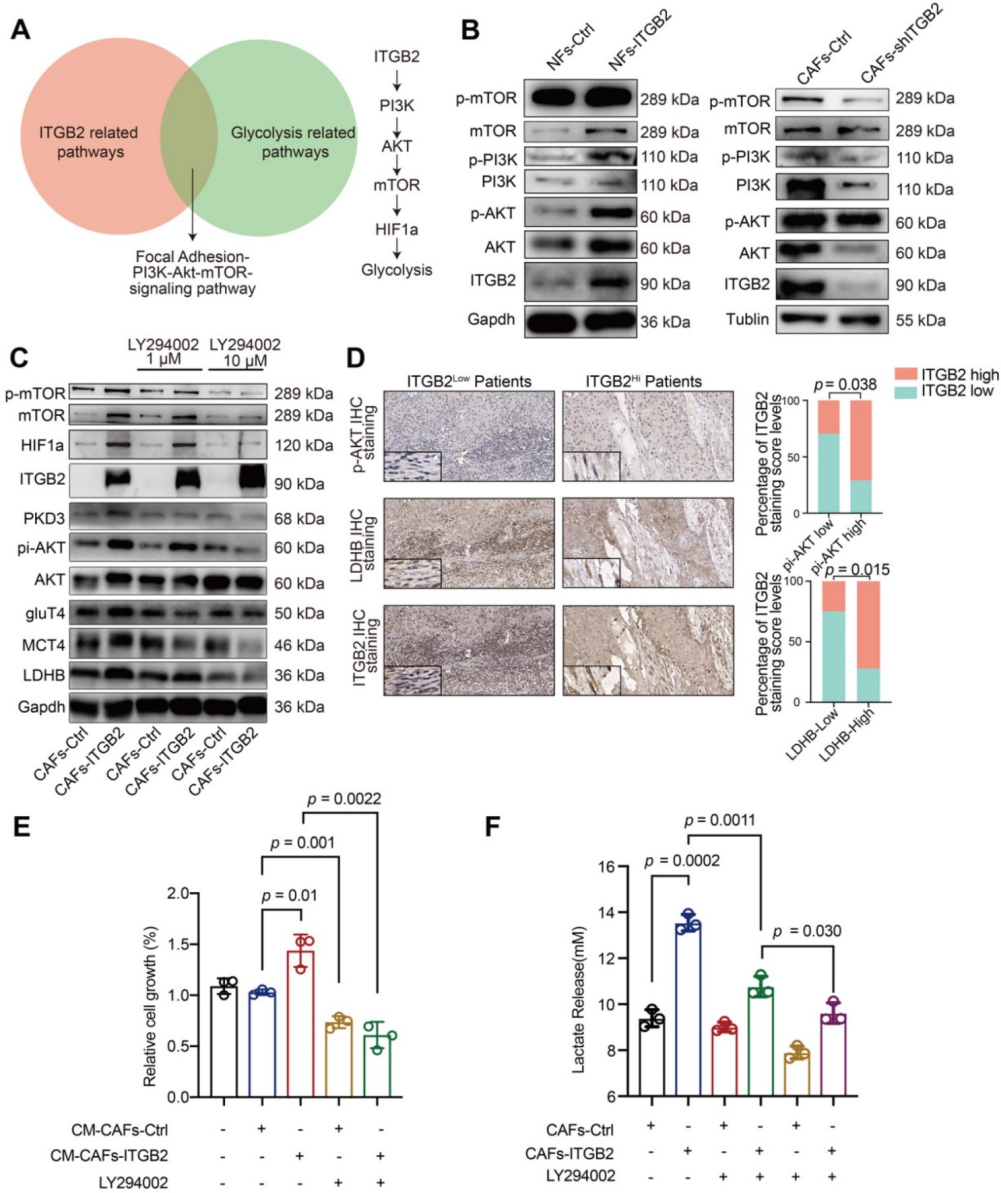
** ITGB2 regulates glycolysis *via* PI3K/AKT/mTOR pathways in CAFs. A)** Venn diagram showing the overlap between ITGB2 related pathways and glycolysis related pathways (Left). Model depicting the pathways of glycolysis induction following ITGB2 upregulation in CAFs (Right). **B)** Western blot showing phosphorylated PI3K, AKT, mTOR, Gapdh and Tublin expression in NFs-Ctrl versus NFs-ITGB2 and CAFs-Ctrl versus CAFs-shITGB2. **C)** Western blot showing phosphorylated PI3K, AKT, mTOR and key glycolytic enzyme expression in CAFs-Ctrl and CAFs-ITGB2 after treated with 1 µM or 10 µM LY294002 for 24 hours. **D)** Representative images of phosphorylated AKT and LDHB expression in ITGB2-low and ITGB2-high CAFs patients (Left). Correlation between ITGB2 and phosphorylated AKT (Upper), LDHB (Down) expression levels in 34 clinical samples. N (ITGB2-low group) = 17, N (ITGB2-high group) = 17. **E)** Proliferation of HSC3 at 24 hours cultured in CM-CAFs-Ctrl or CM-CAFs-ITGB2 treated with or without LY294002. N=3. **F)** Lactate levels of the medium of CAFs-Ctrl or CAFs-ITGB2 treated with or without 1 µM or 10 µM LY294002. N=3. Individual *p* value was shown in each figure.

We then explored the molecular link between ITGB2 expression and glycolytic adaption. Although the WikiPathway database showed that 14 pathways were related to ITGB2 (Table S3), only the PI3K/AKT/mTOR pathway was also involved in the glycolytic pathway (Fig. 5A). Next, we assessed whether ITGB2 regulated glycolysis in CAFs *via* PI3K/AKT/mTOR pathways. As shown in Fig. 5B, the protein levels of pi-PI3K, pi-AKT and pi-mTOR increased when ITGB2 was overexpressed in NFs and decreased when ITGB2 was silenced in CAFs, showing a close relationship between ITGB2 expression and PI3K/AKT/mTOR pathways. To further prove the role of the PI3K/AKT/mTOR pathways in glycolysis regulation *via* ITGB2, we constructed CAFs-ITGB2, which consistently expressed ITGB2. ITGB2 overexpression in CAFs led to enhanced expression of pi-PI3K, pi-AKT, pi-mTOR and the aforementioned key glycolytic enzymes. However, the correspondence increase of the key glycolytic enzymes was abrogated by the PI3K inhibitor LY294002 in a dose dependent manner (Fig. 5C). Accordingly, CAFs treated with LY294002 were unable to produce more lactate and promote HSC3 proliferation even when ITGB2 was overexpressed (Fig. 5E, 5F). All the results demonstrated that ITGB2 regulated glycolysis *via* PI3K/AKT/mTOR pathways in CAFs. Then we reexamined the OSCC samples. The IHC analyses of LDHB and pi-AKT expression in 34 OSCC specimens also showed that higher ITGB2 expression levels were correlated with higher pi-AKT and LDHB expression (Fig. 5D), further supporting the critical role of PI3K/AKT/mTOR signaling in ITGB2-mediated glycolysis.

### Lactate secreted from ITGB2-expressing CAFs promotes OSCC proliferation

**Figure 6:**
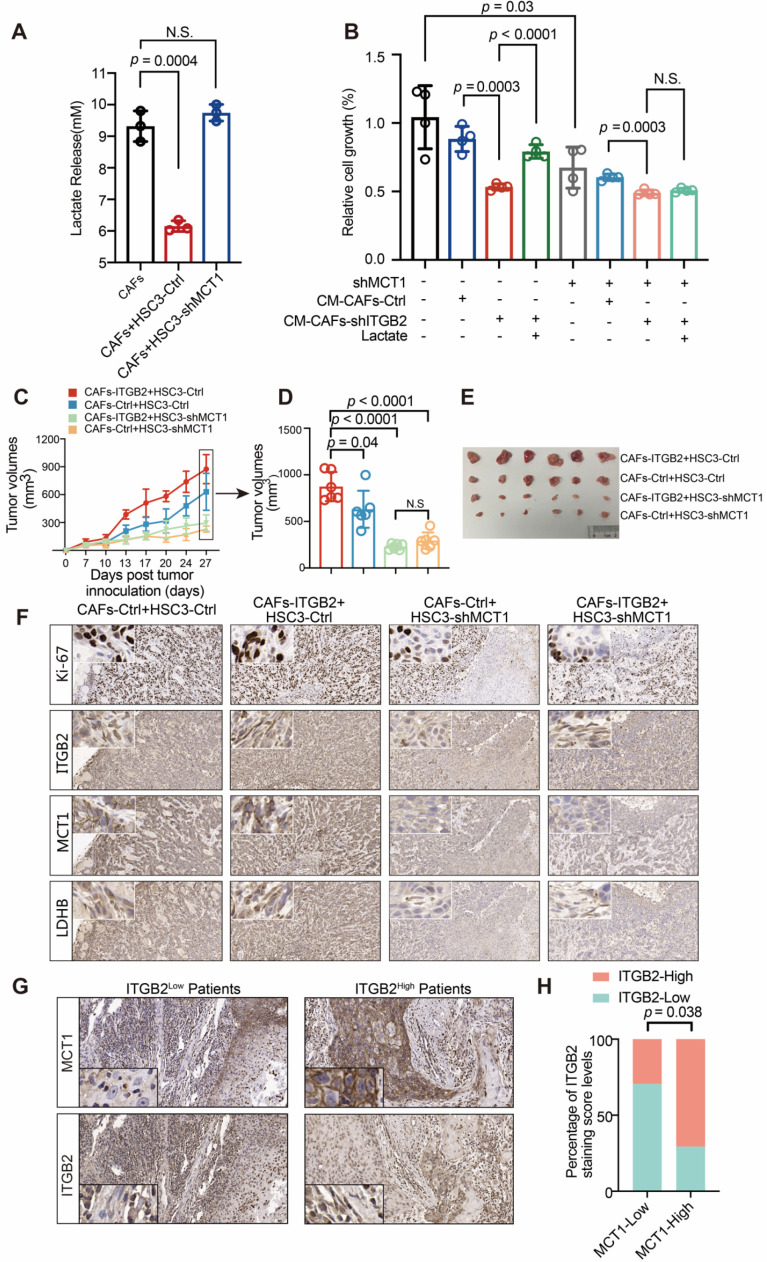
** Lactate secreted by CAFs promotes OSCC proliferation *via* MCT1 expression. A)** Lactate levels in CAFs medium with or without incubation of HSC3-Ctrl or HSC3-shMCT1. N=3. **B)** Proliferation of HSC3-Ctrl or HSC3-shMCT1 at 24 hours in CM-CAFs-Ctrl or CM-CAFs-shITGB2 with or without lactate. N=4. **C)** Tumor growth curves of mice injected with HSC3 cells and CAFs. N=6 mice per group. **D)** Tumor volumes of mice 27 days post tumor inoculation. **E)** Photographs of tumors in nude mice 27 days post tumor implantation. **F)** Representative IHC images of Ki67, ITGB2, MCT1 and LDHB in subcutaneous xenografts. **G)** Representative IHC images of MCT1 expression in ITGB2 low or high expression CAFs of OSCC patients. **H)** Correlation between ITGB2 and MCT1 expression in 34 OSCC patients. N (ITGB2-low group) =17, N (ITGB2-high group) =17.

Given that lactate is the major end product of glycolysis, we next investigated whether lactate was responsible for the pro-proliferative ability of ITGB2-expressing CAFs. As shown in Fig. 6A, extracellular lactate levels dropped when HSC3 cells were in CM, suggesting that lactate was utilized by cancer cells. To verify this, we constructed HSC3-shMCT1, silencing the lactate transporter MCT1 expression (Fig. S6). The results showed that extracellular lactate levels remained unchanged compared with the CM without cell incubation when MCT1 was silenced. Next, we hypothesized that whether the absorbed lactate might be linked to OSCC proliferation. The relative cell growth was lower when HSC3 cells were incubated in the CM-CAFs-shITGB2 where lactate was relatively lower (Fig. 6B). However, adding lactate reversed the decreased cell growth rates of cells in CM-CAFs-shITGB2, suggesting the lactate secreted by CAFs played a pivotal role in OSCC proliferation. We then tested it by using HSC3-shMCT1 cells. Knocking down MCT1 in HSC3 delayed cell growth. Although incubation in CM-CAFs-shITGB2 also retarded the cell proliferation rates, adding lactate failed to recover the proliferation rate, indicating metabolites other than lactate which are transferred by MCT1 may also regulate OSCC proliferation (Fig. 6B)*.* Therefore, in order to prove lactate secreted from ITGB2-expressing CAFs was responsible for the increased proliferation rates of OSCC, we silenced LDHB, which previously had been found to be overexpressed and to produce lactate in ITGB2 expressing CAFs. When LDHB was silenced in CAFs, lactate was significantly down regulated even in ITGB2 overexpressing CAFs (Fig. S7A, S7B). Meanwhile, the pro-proliferative effects of ITGB2 overexpressing CAFs were abrogated when LDHB was silenced. However, adding lactate partially reversed the impaired pro-proliferative effects of silencing LDHB expression in ITGB2 over-expressing CAFs (Fig. S7C). In addition, we further silenced MCT4, which was reported to export lactate in ITGB2-expressing CAFs. As shown in Fig. S7D and S7E, when MCT4 was silenced, extracellular lactate was significantly lower, and cells cultured in CM-CAFs-ITGB2-shMCT4 grew relatively slower than in CM-CAFs-ITGB2. However, adding lactate in CM-CAFs-ITGB2-shMCT4 promoted cell growth (Fig. S7F). These findings indicated that lactate released from ITGB2 expressing CAFs promoted OSCC proliferation.

*In vivo*, the effect of lactate from ITGB2 expressing CAFs was assessed by using a heterotopic oral cancer model. Whereas the tumors of the CAFs-ITGB2 and HSC3-Ctrl group grew more quickly and were larger than the CAFs-Ctrl and HSC3-Ctrl group, the pro-proliferative effects of CAFs-ITGB2 were impaired when MCT1 was silenced in HSC3 (Fig. 6-E). Molecular analyses of the resected tumors confirmed ITGB2 or MCT1 was overexpressed or silenced in the corresponding group (Fig. S8). IHC analyses of tumor tissues revealed that the expression of Ki67, MCT1 in tumor cells and LDHB in CAFs was enhanced in cancer cells when ITGB2 was overexpressed in CAFs (Fig. 6F). Notably, silencing MCT1 abolished the enhanced expression of Ki67 (Fig. 6F). Similarly, the IHC analysis of 34 patients with OSCC also showed that higher ITGB2 expression in CAFs was associated with higher MCT1 expression in HSC3 (Fig. 6G-H). Collectively, the above results showed that lactate secreted by ITGB2 expressing CAFs was absorbed by tumor cells to promote tumor cell proliferation.

### NADH generated from lactate oxidation fuels mitochondria and promotes OSCC proliferation

**Figure 7:**
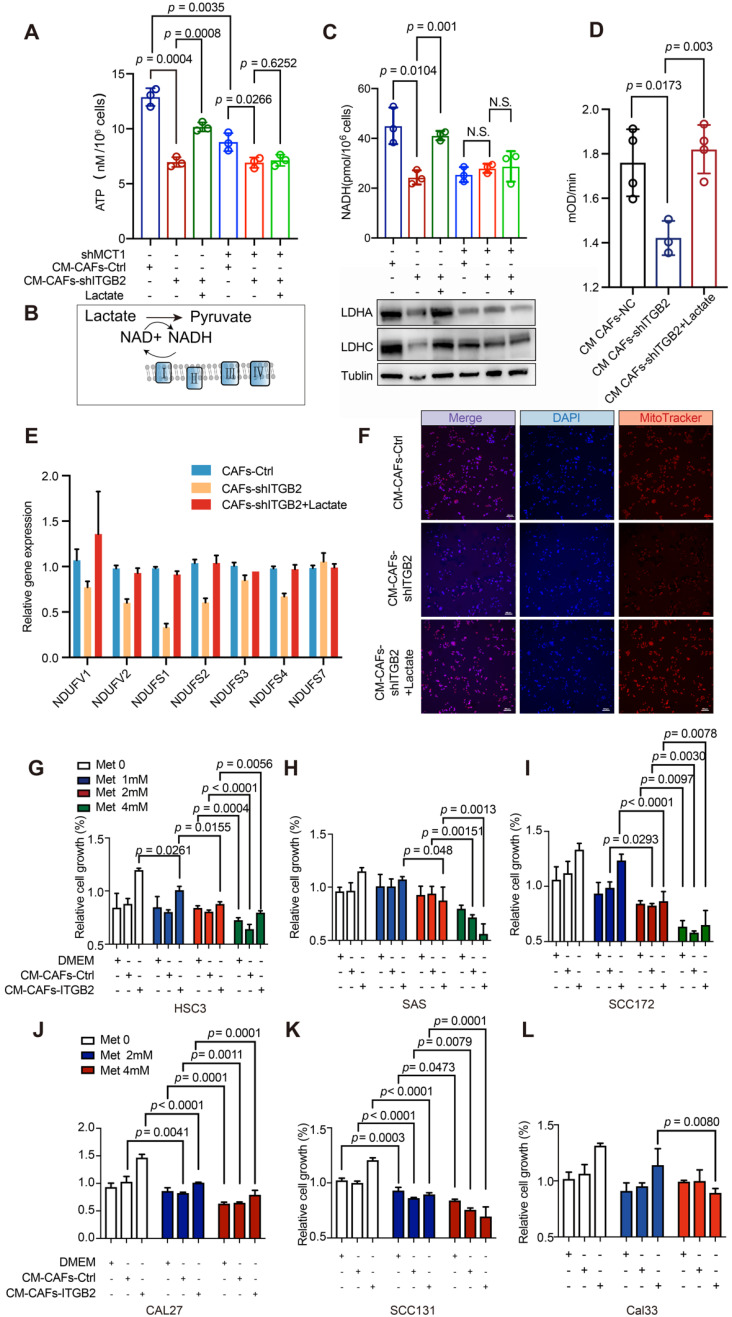
** ITGB2-expressing CAFs promotes OSCC proliferation *via* NADH generated from lactate oxidization. A)** ATP levels of HSC3 or HSC3-shMCT1 cells at 24 hours cultured in CM-CAFs-Ctrl or CM-CAFs-shITGB2 with or without lactate. N=3. **B)** Graphical presentation of lactate oxidization pathway. **C)** Upper: NADH levels of HSC3 or HSC3-shMCT1 cells at 24 hours cultured in CM-CAFs-Ctrl or CM-CAFs-shITGB2 with or without lactate. N=3. Down: Western blot showing LDHA, LDHC and Tublin expression of HSC3 or HSC3-shMCT1 cell lysates at 24 hours cultured in CM-CAFs-Ctrl or CM-CAFs-shITGB2 with or without lactate. **D)** Analyses of mitochondrial complex I activity of HSC3 cells cultured in CM-CAFs-Ctrl or CM-CAFs-shITGB2 with or without lactate. N=4. **E)** mRNA expression levels (relative to Gapdh) of the genes encoding mitochondrial complex I in HSC3 cells cultured in CM-CAFs-Ctrl, CM-CAFs-shITGB2 or CM-CAFs-shITGB2 plus lactate. N=3. **F)** Representative immunofluorescence images of mitochondrial mass (MitoTracker) of HSC3 cells at 24 hours cultured in CM-CAFs-Ctrl or CM-CAFs-shITGB2 with or without lactate. **G-L)** Relative cell growth of HSC3 cells (G), SAS cells (H), SCC172 (I), CAL27 (J), SCC131 (K) and CAL33 (L) cultured in CM-CAFs-Ctrl or CM-CAFs-shITGB2 with or without lactate. N=3. Individual *p* value was shown in each figure.

Although previous studies have reported that lactate “fuels” proliferation, the function and mechanism of lactate, especially secreted by CAFs, in OSCC development remained undiscovered. Here we found that lactate from ITGB2 expressing CAFs provided nearby HSC3 cells with ATP. Less ATP was detected in HSC3 cultured in CM-CAFs-shITGB2, whereas adding lactate reversed these effects (Fig. 7A). To confirm that extracellular lactate was responsible for the different ATP levels, MCT1 was silenced in HSC-3. The results showed although ATP levels of HSC3-shMCT1 cultured in CM-CAFs-Ctrl were still higher than those treated in CM-CAFs-shITGB2, adding lactate to the CAFs-shITGB2 medium failed to recover the decreased ATP levels, further supporting the idea that lactate from CAFs partly promoted ATP generation.

To investigate how lactate was utilized to generate ATP, we focused on NADH, one product of lactate oxidation, because of its importance in cell proliferation (Fig. 7B)*.* HSC3 cells cultured in CM-CAFs-shITGB2 resulted in decreased NADH, which was accompanied by reduced LDHA and LDHC protein expression levels compared with those of the control cells, whereas adding lactate reversed these effects, indicating lactate from ITGB2 expressing CAFs was oxidized by nearby cancer cells and generated NADH (Fig. 7C). Notably, ITGB2 mediated changes in NADH levels, and the lactate metabolizing enzymes of cells were largely abolished when MCT1 was silenced, regardless of the addition of lactate.

Given that NADH was further oxidized in mitochondrial complex I and transferred an electron to generate ATP, we then assessed whether cells in CM-CAFs-shITGB2 exhibited downregulated mitochondrial complex I activity. Cells in CM-CAFs-shITGB2 exhibited attenuated mitochondrial complex I activity, whereas the addition of lactate restored it (Fig. 7D). Consistently, RT-PCR confirmed that ITGB2 mediated lactate release altered some gene expression of mitochondrial complex I in nearby cancer cells (Fig. 7E). Intriguingly, the mitochondrial number was also reduced when HSC3 cells were cultured in CM-CAFs-shITGB2 and the effect was reversed by lactate addition (Fig. 7F). To confirm that NADH is critical for lactate promoted OSCC proliferation, Oxamate, the inhibitor of lactate oxidation, was applied to HSC3 cells to inhibit lactate oxidation 24. As shown in Fig. S9A, the cells in the medium where lactate was the sole carbon were treated with Oxamate at 50 mM for 24 h and displayed reduced ATP levels. Accordingly, when cells were cultured in CM-CAFs-ITGB2 and Oxamate, the growth rates were downregulated. However, the impaired growth rates were reversed by adding NADH (Fig. S6B). Taken together, NADH generated from lactate oxidation promoted OSCC proliferation.

As mitochondrial complex I activation appears to be responsible for ITGB2-mediated growth advantages, we hypothesized whether inhibiting mitochondrial complex I activity might disrupt the promoting proliferation effects of ITGB2-expressing CAFs. The six tested cells displayed different sensitivity to metformin. SCC131 required only 2 mM metformin to partially inhibit proliferation (Fig. 7K). In contrast, the other 5 cell lines were less sensitive to metformin, with 4 mM metformin doses inhibiting proliferation in HSC3, CAL27 and SCC172 or with potential higher doses in CAL33 and SAS. However, when CAL27 and SCC172 cells in CM-CAFs-Ctrl or CM-CAFs-ITGB2 were evaluated, they were more sensitive to metformin and required only 2 mM metformin to inhibit proliferation (Fig. 7-J). Notably, for HSC3, CAL33, SAS, higher ITGB2 expression potentiated inhibitory effects of metformin with lower metformin doses required to inhibit growth rates compared with those in CM-CAFs-Ctrl (Fig. 7G, 7H & 7L). Finally, this indicated that ITGB2 expressing in CAFs mediated OSCC growth relied on mitochondrial complex I activation and displayed a promising therapeutic target.

## Discussion

Although cancer has historically been regarded as a disorder of cell proliferation, increasing evidence suggests that it is a metabolic disease. To meet the bioenergetic, biosynthetic and redox demands of continuous cell growth, the metabolic programs of tumors are rewired. In addition, the infiltrated immune cells, blood vessels and stromal cells which co-exist and interact with tumor cells remodel their metabolic properties accordingly to share or compete for nutrients with cancer 25, 26. For example, CAFs have been reported to undergo the Warburg effect to supply nutrients to nearby cancer cells to support their growth and metastasis 27. This phenomenon is known as reverse the Warburg effect and has been reported in a number of tumor types. However, oxidative stromal fibroblasts and glycolytic cancer cells have been found in a colorectal cancer model, indicating metabolic variety between tumor types 28. Thus, 1) whether the reverse Warburg effect occurred in OSCC; 2) its underling mechanism and 3) implication as the therapeutic target remain elusive. In this study, we revealed the mechanism of ITGB2-induced glycolysis in CAFs in OSCC. Compared with NFs, ITGB2 was found to be upregulated in CAFs and displayed enhanced glycolysis activity and lactate release *via* PI3K/AKT/mTOR pathways. Accordingly, the metabolite lactate derived from ITGB2-CAFs was immediately oxidized, and the generation of ATP was therefore increased by OXPHOS which required NADH, thus fueling OSCC proliferation. Notably, the pro-proliferative activity of ITGB2-expressing CAFs may also provide an advantage for cancer treatment as OSCC cells cultured in ITGB2-expressing CAFs medium render OSCC cells sensitive to metformin.

ITGB2 is one of nine β integrins and is reported to be predominantly expressed on immune cells. It is reported that it is involved in leucocyte extravasation, the binding and removal of fragments of complement, phagocytosis and intracellular killing of pathogenic microbes 20, 21. However, these studies about ITGB2 focused on immune cells. The expression and mechanism of ITGB2 in other types of cells were largely unknown. Liu *et al*. recently reported that YAP induced ITGB2 expression in cancer cells to promote cell invasion through the endothelium in a manner mimicking leukocytes 23. In our study, ITGB2 was also found to be overexpressed in cancer cells, proved by IHC and immunofluorescence analyses of ITGB2 in OSCC patients. Apart from cancer cells, here we showed that ITGB2 was also expressed in CAFs. Compared with NFs, ITGB2 was overexpressed in CAFs in OSCC. In addition, GEO analysis showed that ITGB2 was also overexpressed in prostate cancer, breast cancer and ovarian cancer, indicating it is not an exclusive phenomenon in OSCC. Moreover, the study of 80 patients with OSCC found that only ITGB2 expression in CAFs other than infiltrating immune cells had diagnostic and prognostic values. This indicated that the function of ITGB2 varied between cell types in OSCC. However, the mechanism for regulating ITGB2 expression was undiscovered in our research and deserves further study.

Traditionally, integrins have been viewed as important regulators of cell survival, proliferation, adhesion and migration. However, mounting evidence suggests that integrins also control various metabolic signals and pathways. ITGB1 was reported to be involved in glycolysis shift, amino acids transport and interact with MCT4 to mediate lactate transport 19. Apart from this, in triple negative breast cancer ITGB4 induced glycolysis in CAFs 11. Consistently, here we found that ITGB2 promoted glycolysis *via* PI3K/AKT/mTOR pathways in CAFs. It is known as the reverse Warburg effect and is reported in several types of cancers. In breast cancer, oxidized ATM and ITGB4 were found to enhance glycolysis activity in CAFs 10. In melanoma, TGF-β1 or PDGF induced CAFs to switch from oxidative phosphorylation to anaerobic glycolysis 9. In ovarian cancer, CXCL14 regulated LINC00092 which bound glycolytic enzyme fructose-2,6-biphosphatase (PFKFB2) to promote its metastasis. Despite the reverse metabolic coupling in head and neck cancer found by Kumar *et al.*, our finding showed that the reverse Warburg effect occurred in OSCC *via* ITGB2-mediated glycolysis in CAFs 29.

Although lactate was previously known as a metabolic waste, mounting evidence now suggests it is a major carbon source in normal tissue and tumors 30. In this study, we found that lactate from ITGB2-expressing CAFs was absorbed and metabolized in OSCC to generate NADH to fuel OSCC proliferation. Targeting mitochondrial complex I effectively inhibited the pro-proliferative effects of ITGB2 expressing CAFs, further supporting lactate oxidation in OSCC. Although previous studies have shown multiple other metabolic fluxes of exogenous lactate, which can be utilized to generate lipids, glycogen and renew TCA cycle intermediates 31-33, we did not examine the other lactate fluxes. Therefore, it remains undiscovered whether the absorbed lactate can be used as carbon precursors other than as an oxidative fuel source. In addition, lactate was also a signaling molecular and instructed CAFs to produce hepatocyte growth factor (HGF) 34. In addition, Zhang *et al.* recently found that lactate-derived lactylation of histone lysine residues serves as an epigenetic modification, indicating that lactate plays more roles than previously expected and deserves further study 35.

Metformin is an anti-hyperglycemic agent and is found to be associated with improved cancer outcomes 36, 37. It is reported that metformin decreases mitochondrial respiration by inhibiting the glycerol-phosphate shuttle or directly inhibiting mitochondrial complex I 38. As more NADH was generated in cells cultured in ITGB2-expressing CAFs and mitochondrial complex I activity was enhanced, we suggested metformin might interrupt the pro-proliferative effects of ITGB2-expressing CAFs. Although the tested OSCC cells displayed different sensitivity to metformin, we found that ITGB2 overexpression in CAFs rendered the cultured cells most sensitive to metformin. Given that lactate oxidation consumes NAD^+^ which was recycled from OXPHOS, we suspected it might be because of the unbalanced NAD^+^/NADH ratios for the cells cultured in ITGB2-expressing CAFs which required more NAD^+^ while metformin interrupted NAD^+^ recycling.

In conclusion, our study found that ITGB2 played a critical role in CAFs to promote OSCC proliferation due to enhanced glycolysis activity in CAFs *via* PI3K/AKT/mTOR pathways. ITGB2-expressing CAFs secreted lactate to nearby OSCC cells to generate ATP *via* OXPHOS. Meanwhile, we found that the pro-proliferative effects of ITGB2 was the Achilles' heel which rendered the nearby OSCC cells more sensitive to metformin and was a potential therapeutic target.

## Supplementary Material

Supplementary figures and tables.

## Figures and Tables

**Table 1:** Correlation between clinicopathological characteristics of OSCC patients and ITGB2 expression in FLC and TILs

		FLCs				TILs		
		ITGB2 Low (%)	ITGB2 High (%)	*p*		ITGB2 Low (%)	ITGB2 High (%)	*p*
**Gender**								
Female	45 (56.3%)	18 (40.0%)	27 (60.0%)	0.308	45 (58.4%)	27 (60%)	18 (40%)	0.099
Male	35 (43.8%)	18 (51.4%)	17 (48.6%)		32 (41.6%)	22 (62.9%)	10 (28.6%)	
**Age**								
< 60	38 (47.5%)	17 (44.7%)	21 (55.3%)	0.964	36 (46.8%)	28 (73.7%)	8 (21.1%)	**0.04**
≥ 60	42 (52.5%)	19 (45.2.0%)	23 (54.8%)		41 (53.2%)	21 (50.0%)	20 (46.7%)	
**TNM**								
I-II	62 (77.5%)	33 (53.2%)	29 (46.8%)	0.006*	60 (77.9%)	40 (64.5%)	20 (32.3%)	0.529
III-IV	18 (22.5%)	3 (16.7%)	15 (83.3%)		17 (22.1%)	9 (50.0%)	8 (44.4%)	
**Differentiation**								
Well	52 (65.0%)	25 (48.1%)	27 (51.9%)	0.451	49 (63.6%)	34 (65.4%)	15 (28.8%)	0.162
Moderate/poor	28 (35.0%)	11 (39.3%)	17 (60.7%)		28 (36.4%)	15 (53.6%)	13 (46.4%)	
**WPOI**								
1-3	44 (55.0%)	24 (54.5%)	20 (45.5%)	0.058	42 (54.5%)	29 (65.9%)	13 (29.5%)	0.511
4-5	36 (45.0%)	12 (33.3%)	24 (66.7%)		35 (45.5%)	20 (55.6%)	15 (41.7%)	
**Nerve invasion**								
No	57 (71.3%)	28 (49.1%)	29 (50.9%)	0.438	55 (84.6%)	35 (61.4%)	20 (35.1%)	0.662
Yes	13 (16.3%)	7 (63.6%)	4 (36.4%)		10 (15.4%)	7 (63.6%)	3 (27.3%)	
**DOI**								
< 5 mm	52 (65%)	28 (53.8%)	24 (46.2%)	0.03*	51 (66.2%)	31 (59.6%)	20 (38.5%)	0.388
≥ 5 mm	28 (35%)	8 (28.6%)	20 (71.4%)		26 (33.8%)	18 (64.3%)	8 (23.6%)	

*, represented that differences were considered statistically significant with *P*<0.05. FLCs, fibroblast-like cells; TILs, tumor-infiltrating lymphocytes. WPOI, worst pattern of invasion. DOI, depth of invasion.
