# Supplementary material for: ITGB2-mediated metabolic switch in CAFs promotes OSCC proliferation by oxidation of NADH in mitochondrial oxidative phosphorylation system
Source: Theranostics. 2020 Oct 26;10(26):12044–59. doi: 10.7150/thno.47901 (PMC7667693; doi:10.7150/thno.47901)
Supplement: Supplementary file 1 — Supplementary figures and tables. [file thnov10p12044s1.pdf]

Table S1 RT-PCR primers sequences

ITGB2-F	TGCGTCCTCTCTCAGGAGTG	PKLR-F	TCAAGGCCGGGATGAACATTG
ITGB2-R	GGTCCATGATGTCGTCAGCC	PKLR-R	CTGAGTGGGGAACCTGCAAAG
CD45-F	ACCACAAGTTTACTAACGCAAGT	PKM-F	ATGTCGAAGCCCCATAGTGAA
CD45-R	TTTGAGGGGGATTCCAGGTAAT	PKM-R	TGGGTGGTGAATCAATGTCCA
GluT4-F	TGGGCGGCATGATTTCCTC	LDHB-F	TGGTATGGCGTGTGCTATCAG
GluT4-R	GCCAGGACATTGTTGACCAG	LDHB-R	TTGGCGGTCACAGAATAATCTTT
GluT2-F	GCTGCTCAACTAATCACCATGC	LDHA-F	ATGGCAACTCTAAAGGATCAGC
GluT2-R	TGGTCCCAATTTTGAAAACCCC	LDHA-R	CCAACCCCAACAACTGTAATCT
GluT1-F	GGCCAAGAGTGTGCTAAAGAA	PDK3-F	CGCTCTCCATCAAACAATTCCT
GluT1-R	ACAGCGTTGATGCCAGACAG	PDK3-R	CCACTGAAGGGCGGTTAAGTA
GluT3-F	GCTGGGCATCGTTGTTGGA	PDK4-F	GGAGCATTTCTCGCGCTACA
GluT3-R	GCACTTTGTAGGATAGCAGGAAG	PDK4-R	ACAGGCAATTCTTGTCGCAAA
HK1-F	GCTCTCCGATGAAACTCTCATAG	PDK1-F	CTGTGATACGGATCAGAAACCG
HK1-R	GGACCTTACGAATGTTGGCAA	PDK1-R	TCCACCAAACAATAAAGAGTGCT
HK2-F	GAGCCACCACTCACCCTACT	HIF1a-F	TTCCCGACTAGGCCCATTC
HK2-R	CCAGGCATTCGGCAATGTG	HIF1a-R	CAGGTATTCAAGGTCCCATTTCA
HK3-F	GGACAGGAGCACCCTCATTTC	CS-F	TGCTTCCTCCACGAATTTGAAA
HK3-R	CCTCCGAATGGCATCTCTCAG	CS-R	CCACCATACATCATGTCCACAG
PFKL-F	GCTGGGCGGCACTATCATT	ACO2-F	CCCTACAGCCTACTGGTGACT
PFKL-R	TCAGGTGCGAGTAGGTCCG	ACO2-R	TGTACTCGTTGGGCTCAAAGT
PFKM-F	GGTGCCCGTGTCTTCTTTGT	ACO1-F	AACCCATTCGCACACCTTG
PFKM-R	AAGCATCATCGAAACGCTCTC	ACO1-R	ATGGTAAGCGCCCATATCTTG
PFKP-F	GCATGGGTATCTACGTGGGG	IDH1-F	TGTGGTAGAGATGCAAGGAGA
PFKP-R	CTCTGCGATGTTTGAGCCTC	IDH1-R	TTGGTGACTTGGTCGTTGGTG
PGK1-F	TGGACGTTAAAGGGAAGCGG	IDH2-F	CGCCACTATGCCGACAAAAG
PGK1-R	GCTCATAAGGACTACCGACTTGG	IDH2-R	ACTGCCAGATAATACGGGTCA
PGK2-F	AAACTGGATGTTAGAGGGAAGCG	IDH3a-F	CCCGCGTGGATCTCTAAGG
PGK2-R	GGCCGACCTAGATGACTCATAAG	IDH3a-R	AATTTCTGGGCCAATACCATCTC
ENO1-F	AAAGCTGGTGCCGTTGAGAA	IDH3b-F	GAGCCAAGTCTCAGCGGATT
ENO1-R	GGTTGTGGTAAACCTCTGCTC	IDH3b-R	GGGCATCACAAGCACATCAAA
ENO2-F	AGCCTCTACGGGCATCTATGA	IDH3g-F	AAACAATTCCTCCGTCCGCTA
ENO2-R	TTCTCAGTCCCATCCAACTCC	IDH3g-R	ATGGCATTGCGAATGTCCTCT
ENO3-F	GGCTGGTTACCCAGACAAGG	OGDHL-F	CATCGACAAATCCAGCGAGAT
ENO3-R	TCGTACTTCCCATTGCGATAGAA	OGDHL-R	ATCCTCTCATGGTACATGCCC

Table S2

AccID	Case	Control	Log2FC	FDR	5# CAFs	1# CAFs	2# CAFs	3# CAFs	4# CAFs	1# NFs	3# NFs	4# NFs	5# NFs	2# NFs
ITGB2	142.56	5.03	4.82	0.03	327	16	19	226	167	3	15	2	2	49

Table S3

ITGB2 related pathways	
1	Mechanoregulation and pathology of YAP/TAZ via Hippo and non-Hippo mechanisms (Homo sapiens)
2	Cells and Molecules involved in local acute inflammatory response (Homo sapiens)
3	TYROBP Causal Network (Homo sapiens)
4	Microglia Pathogen Phagocytosis Pathway (Homo sapiens)
5	Hippo-Merlin Signaling Dysregulation (Homo sapiens)
6	Integrin-mediated Cell Adhesion (Homo sapiens)
7	Integrin cell surface interactions (Homo sapiens)
8	Toll-like Receptor Cascades (Homo sapiens)
9	Cell surface interactions at the vascular wall (Homo sapiens)
10	Neutrophil degranulation (Homo sapiens)
11	Viral Acute Myocarditis (Homo sapiens)
12	Interleukin-4 and Interleukin-13 signaling (Homo sapiens)
13	Immunoregulatory interactions between a Lymphoid and a non-Lymphoid cell (Homo sapiens)
14	Focal Adhesion-PI3K-Akt-mTOR-signaling pathway (Homo sapiens)

Glycolysis related pathways	
1	Glycolysis and Gluconeogenesis - for workshop (Homo sapiens)
2	Glycolysis and Gluconeogenesis (Homo sapiens)
3	Computational Model of Aerobic Glycolysis (Homo sapiens)
4	Computational Model of Aerobic Glycolysis - Augmented (Homo sapiens)
5	Glycolysis Pathway D (2) (Homo sapiens)
6	HIF1A and PPARG regulation of glycolysis (Homo sapiens)
7	WikiPathways Academy: fig-met-1-second (Homo sapiens)
8	Polyol Pathway (Homo sapiens)
9	WikiPathways Academy: fig-met-1-third (Homo sapiens)
10	WikiPathways Academy: fig-met-1-fourth (Homo sapiens)
11	Acetylcholine Synthesis (Homo sapiens)
12	Serine Metabolism (Homo sapiens)
13	SCFA and skeletal muscle substrate metabolism (Homo sapiens)
14	Synthesis and Degradation of Ketone Bodies (Homo sapiens)
15	Glucose Homeostasis (Homo sapiens)
16	Pentose Phosphate Metabolism (Homo sapiens)

17	WikiPathways Academy: fig-met-1-last (Homo sapiens)
18	Transcription factor regulation in adipogenesis (Homo sapiens)
19	Photodynamic therapy-induced HIF-1 survival signaling (Homo sapiens)
20	Nanoparticle triggered regulated necrosis (Homo sapiens)
21	fig-met-1-last-solution (Homo sapiens)
22	aspirin and miRNAs (Homo sapiens)
23	Sleep regulation (Homo sapiens)
24	NAD+ metabolism (Homo sapiens)
25	TCA Cycle and Deficiency of Pyruvate Dehydrogenase complex (PDHc) (Homo sapiens)
26	Hereditary leiomyomatosis and renal cell carcinoma pathway (Homo sapiens)
27	Ketogenesis and Ketolysis (Homo sapiens)
28	Major receptors targeted by epinephrine and norepinephrine (Homo sapiens)
29	Glycogen Synthesis and Degradation (Homo sapiens)
30	Pathways in clear cell renal cell carcinoma (Homo sapiens)
31	AMP-activated Protein Kinase (AMPK) Signaling (Homo sapiens)
32	Alanine and aspartate metabolism (Homo sapiens)
33	Metabolic reprogramming in colon cancer (Homo sapiens)
34	Glycerophospholipid Biosynthetic Pathway (Homo sapiens)
35	Focal Adhesion-PI3K-Akt-mTOR-signaling pathway (Homo sapiens)
36	Tryptophan metabolism (Homo sapiens)
37	Angiopoietin Like Protein 8 Regulatory Pathway (Homo sapiens)
38	Amino Acid metabolism (Homo sapiens)
39	Human metabolism overview (Homo sapiens)
40	PI3K-Akt Signaling Pathway (Homo sapiens)

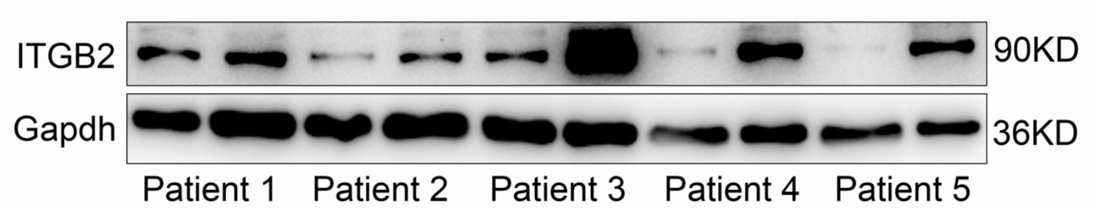

Figure S1 Western blot analysis of ITGB2 (ab53009, Abcam) expression in NFs and CAFs.

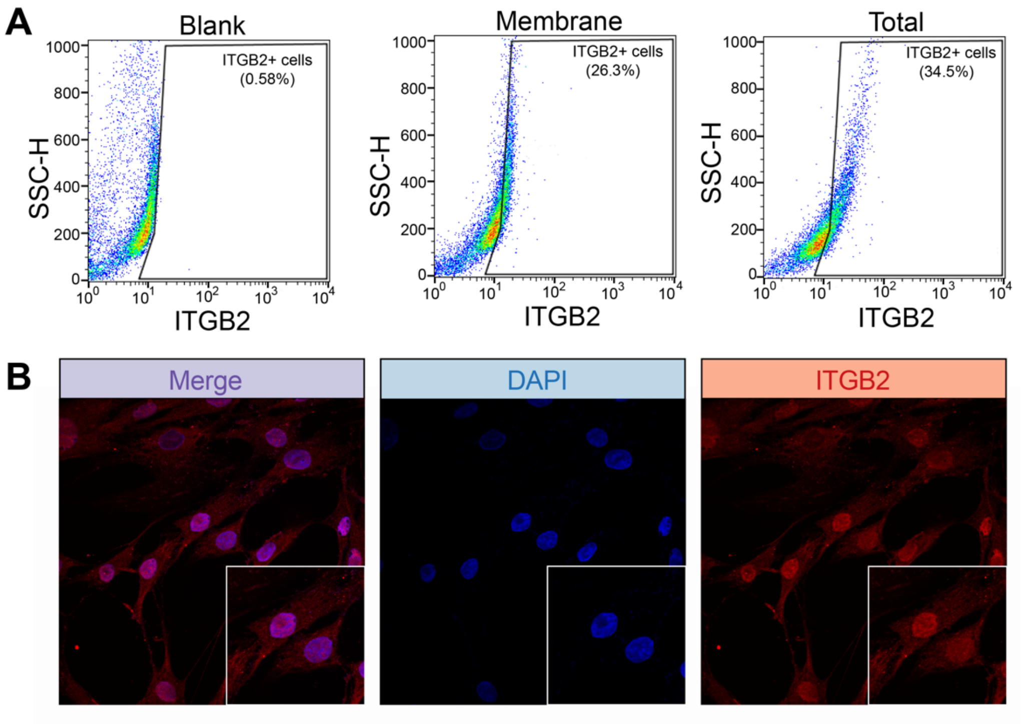

Figure S2 A) Flow cytometry analysis of total and membrane ITGB2 expression in CAFs. B) IFC analysis of ITGB2 expression in CAFs.

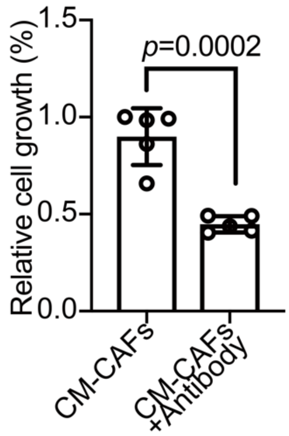

Figure S3 CCK8 analysis of HSC3 cells cultured in CM-CAFs and CM-CAFs+Antibody.

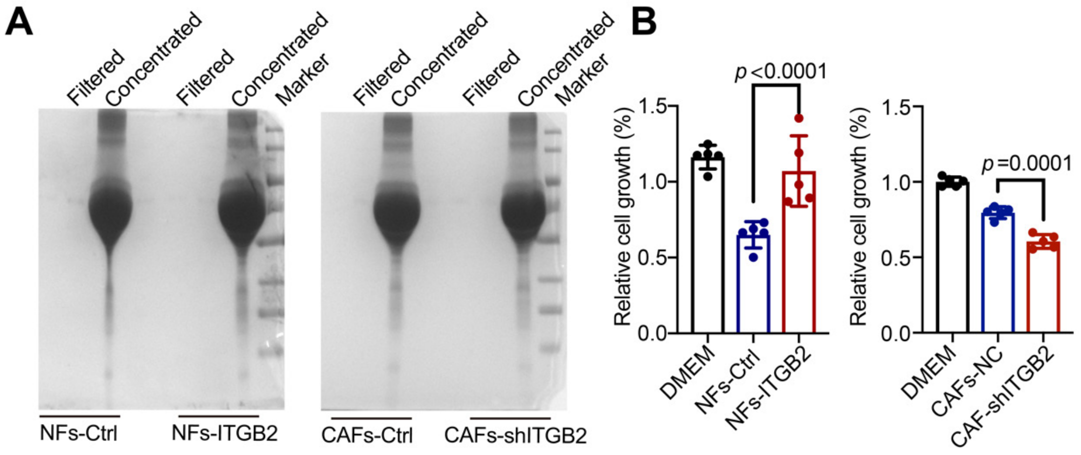

Fig. S4 A) Coomassie stain of the medium passing through 3 kDa cut-off filter and concentrated medium of NFs-Ctrl, NFs-ITGB2 (Left), CAFs-Ctrl and CAFs-shITGB2 (Right). B) The relative cell growth between NFs-Ctrl and NFs-ITGB2 (Left) and CAFs-Ctrl and CAFs-shITGB2 (Right).

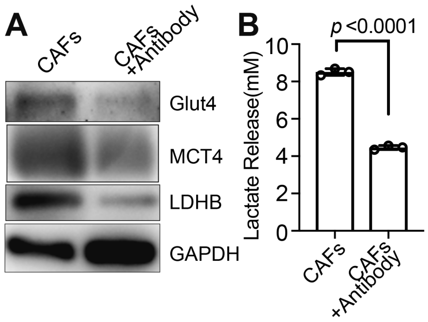

Fig. S5 A) Western blot analysis of glycolytic proteins of CAFs treated with or without ITGB2 neutralizing antibody. B) Lactate levels of CAFs treated with or without ITGB2 neutralizing antibody.

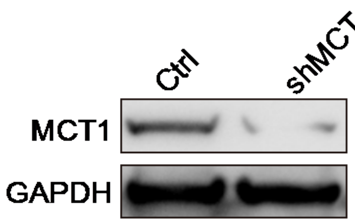

Fig. 6 Western blot analysis of MCT1 expression.

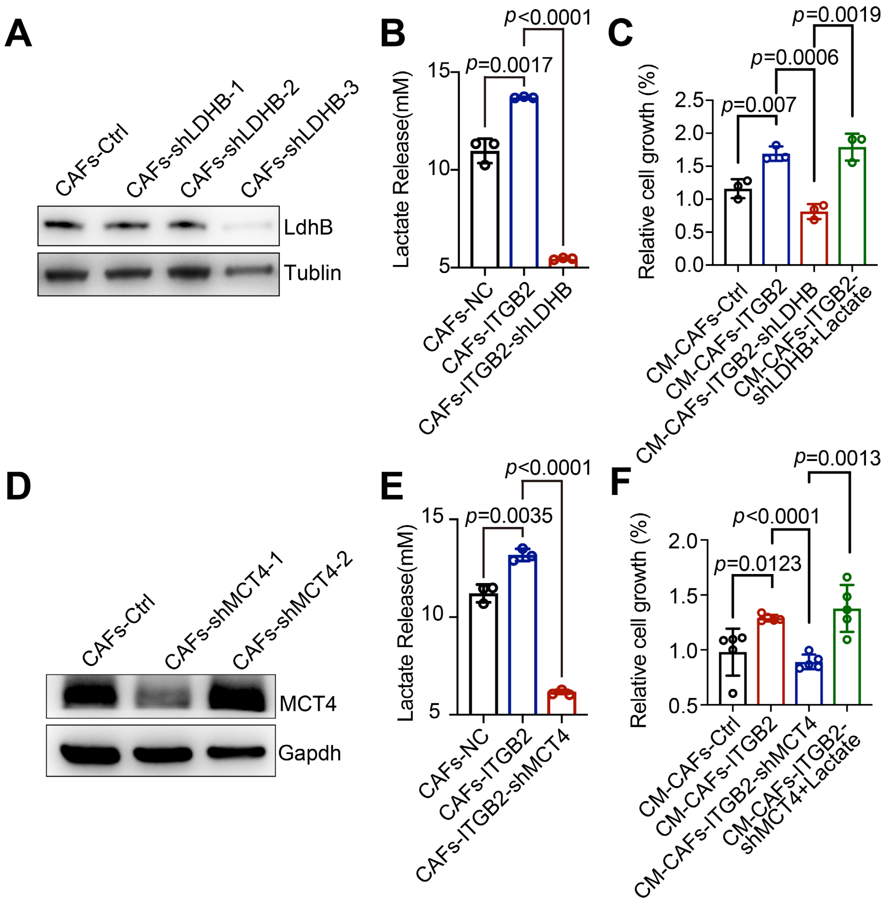

Figure S7 A) Western blot analysis LDHB expression in CAFs. B) Lactate release in culture medium of CAFs-NC, CAFs-ITGB2 and CAFs-ITGB2-shLDHB. C) Relative cell growth of HSC3 cells in CM-CAFs-Ctrl, CM-CAFs-ITGB2, CM-CAFs-ITGB2-shLDHB and CM-CAFs-ITGB2-shLDHB plus lactate. D) Western blot analysis MCT4 expression in CAFs. B) Lactate release in culture medium of CAFs-NC, CAFs-ITGB2 and CAFs-ITGB2-shMCT4. C) Relative cell growth of HSC3 cells in CM-CAFs-Ctrl, CM-CAFs-ITGB2, CM-CAFs-ITGB2-shMCT4 and CM-CAFs-ITGB2-shMCT4 plus lactate.

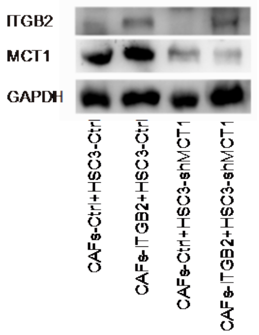

Figure S8 Western blot showing protein levels of ITGB2, MCT1 and GAPDH of tumor lysates from mice in CAFs-Ctrl+HSC3-Ctrl, CAFs-ITGB2+HSC3-Ctrl, CAFs-Ctrl+HSC3-shMCT1, CAFs-ITGB2+HSC3-shMCT1.

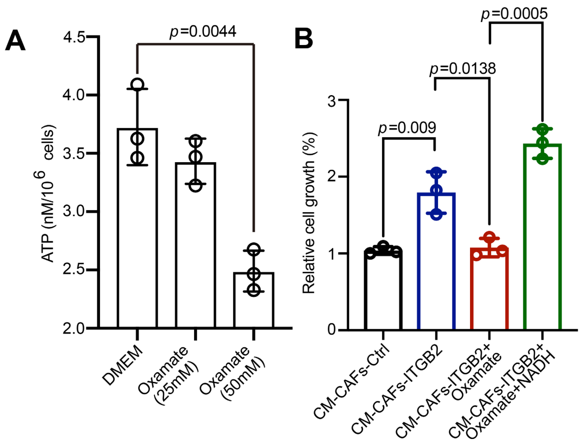

Figure S9 A) ATP levels of cells cultured in DMEM (contain lactate) without glutamine and glucose treated with or without Oxamate. B) Relative cell growth of cells cultured in CM-CAFs-Ctrl, CM-CAFs-ITGB2, CM-CAFs-ITGB2 plus Oxamate and CM-CAFs-ITGB2 plus Oxamate and NADH.
